# Women at Michigan University

**Published:** 1876-02

**Authors:** 


### WOMEN AT MICHIGAN UNIVERSITY.

Having been in happy operation for twenty-nine years as a university exclusively for men, in the year 1870 it opened its doors in all departments for the admission of women. According to the most recent returns, one hundred and seventeen of that sex are now availing themselves of the right to university instruction thus recognized. In the distribution which they have made of themselves among the several departments, there is no little significance--four of them having chosen the law, forty-seven medicine and sixty-six literature and science. Before 1870 there were several colleges in America which had adopted the system of co-education; but all of these had adopted that system from the beginning. Michigan is the first university which, having begun its life and attained eminent success upon the old and exclusive system, then deliberately incorporated upon itself the new and more comprehensive plan. The resolution to do so was by no means a hasty one, or taken with much cheerfulness. It had been under consideration for twenty years and when adopted at last, it was adopted with no little anxiety. Our experience of five years has, I think, convinced everybody here that this anxiety was not well founded. Neither good order nor the scholarship of the University has suffered any harm from the presence of ladies in its class-rooms; while the physical disasters to the women themselves, which an eminent medical authority has of late clearly demonstrated to be the penal consequences of feminine toil at the dry and arduous tasks of university study, have thus far strangely failed to make their appearance in this neighborhood. Indeed, the ladies here seem to thrive ludicrously well under the rugged regimen to which they have been put; and their omission to verify the predictions of an *a priori* alarm is something bordering upon the cruel. A benevolent mind observing these things can hardly do less than utter a word of kindly caution to all persons who still desire to take unalloyed comfort in the doctrine that women are not fit for universities, or that universities are not fit for women; such persons should abjure the neighborhood of iustitutions like the University of Michigan, and faithfully limit themselves to speculative data. —*Scribner for February.*

